# Mitochondrial Protein PGAM5 Regulates Mitophagic Protection against Cell Necroptosis

**DOI:** 10.1371/journal.pone.0147792

**Published:** 2016-01-25

**Authors:** Wei Lu, Junhui Sun, Jeong Seon Yoon, Yan Zhang, Lixin Zheng, Elizabeth Murphy, Mark P. Mattson, Michael J. Lenardo

**Affiliations:** 1 Molecular Development of the Immune System Section, Laboratory of Immunology, National Institute of Allergy and Infectious Diseases, National Institutes of Health, Bethesda, Maryland, United States of America; 2 Systems Biology Center,National Heart, Lung, and Blood Institute, National Institutes of Health, Bethesda, Maryland, United States of America; 3 Cellular and Molecular Neuroscience Section, Laboratory of Neuroscience, National Institute on Aging, National Institutes of Health, Baltimore, Maryland, United States of America; 4 Molecular Mechanism of Apoptosis Section, Cell and Cancer Biology Branch, National Cancer Institute, National Institutes of Health, Bethesda, Maryland, United States of America; Toho University School of Medicine, JAPAN

## Abstract

Necroptosis as a molecular program, rather than simply incidental cell death, was established by elucidating the roles of receptor interacting protein (RIP) kinases 1 and 3, along with their downstream partner, mixed lineage kinase-like domain protein (MLKL). Previous studies suggested that phosphoglycerate mutase family member 5 (PGAM5), a mitochondrial protein that associates with RIP1/RIP3/MLKL complex, promotes necroptosis. We have generated mice deficient in the pgam5 gene and surprisingly found PGAM5-deficiency exacerbated rather than reduced necroptosis in response to multiple *in vitro and in vivo* necroptotic stimuli, including ischemic reperfusion injury (I/R) in the heart and brain. Electron microscopy, biochemical, and confocal analysis revealed that PGAM5 is indispensable for the process of PINK1 dependent mitophagy which antagonizes necroptosis. The loss of PGAM5/PINK1 mediated mitophagy causes the accumulation of abnormal mitochondria, leading to the overproduction of reactive oxygen species (ROS) that worsen necroptosis. Our results revise the former proposal that PGAM5 acts downstream of RIP1/RIP3 to mediate necroptosis. Instead, PGAM5 protects cells from necroptosis by independently promoting mitophagy. PGAM5 promotion of mitophagy may represent a therapeutic target for stroke, myocardial infarction and other diseases caused by oxidative damage and necroptosis.

## Introduction

Programmed cell death is morphologically classified into apoptosis, autophagic cell death, programmed necrosis/necroptosis, and mitotic catastrophe [1–4]. Necroptosis as a molecular program, rather than simply incidental cell death, was established by elucidating the roles of receptor interacting protein (RIP) kinases 1 and 3 [5–10]. Following tumor necrosis factor (TNF)-α stimulation, RIP3 is phosphorylated by RIP1 to initiate a molecular cascade that has been proposed to involve mixed lineage kinase domain-like protein (MLKL) and phosphoglycerate mutase family member 5 (PGAM5) [11–14]. These proteins were proposed to form oligomers on the plasma membrane as well as intracellular and organelle membranes that disrupt their integrity and cause necroptosis [11–14].

PGAM5 is a 32-kDa mitochondrial membrane protein with homology to a family of phosphoglycerate mutases but lacking similar enzymatic function [15]. It serves as an anti-oxidant regulator in the Kelch ECH associating protein 1-nuclear factor-E2-related factor 2 (KEAP1-NRF2) signaling pathway and as a serine/threonine phosphatase that regulates ASK1 kinase activity [15–17]. It has been reported that PGAM5 is the anchor of RIP1-RIP3-MLKL complex on mitochondria of cancer cells [12]. However, most of the PGAM5 studies were done in tumor cell lines and therefore the physiological function of PGAM5 *in vivo* remains unclear. Recent studies have shown that PGAM5 is critical for mitochondria homeostasis by promoting mitophagy [18–20]. PGAM5 can help stabilize the mitophagy-inducing protein PINK1 on damaged mitochondria. PGAM5 deficiency disrupts PINK1-mediated mitophagy and led to a Parkinson’s-like phenotype involving dopaminergic neurodegeneration and mild dopamine loss in a Pgam5-deficient mouse model *in vivo* [18].

ROS generated and leaking from mitochondria, especially those from stressed or damaged mitochondria caused by multiple necroptotic stimuli, result in oxidative damage to proteins and lipids that can lead to necroptosis [10, 21]. It has been suggested that a broad range of chronic disorders including ischemic cardiovascular disease [22] and neurological disorders such as stroke and Parkinson’s disease may involve ROS related necroptosis [23]. However, the molecular regulation of necroptosis through mitochondria in these conditions is not fully understood.

In our present study, we employed *in vitro* and *in vivo* assays to investigate the physiological function of PGAM5 in necroptosis using the knockout mouse strain we have generated [18]. We found PGAM5 actually protects cells from necroptosis through promoting mitophagy, which is a selective form of autophagy that targets unhealthy mitochondria for lysosomal recycling and prevents ROS overproduction. Our study suggests that PGAM5 provides a potential link between malfunctions of mitophagy and the pathogenesis of necroptosis.

## Results

### PGAM5 is an important protective gene for ischemic injury

**Fig 1:**
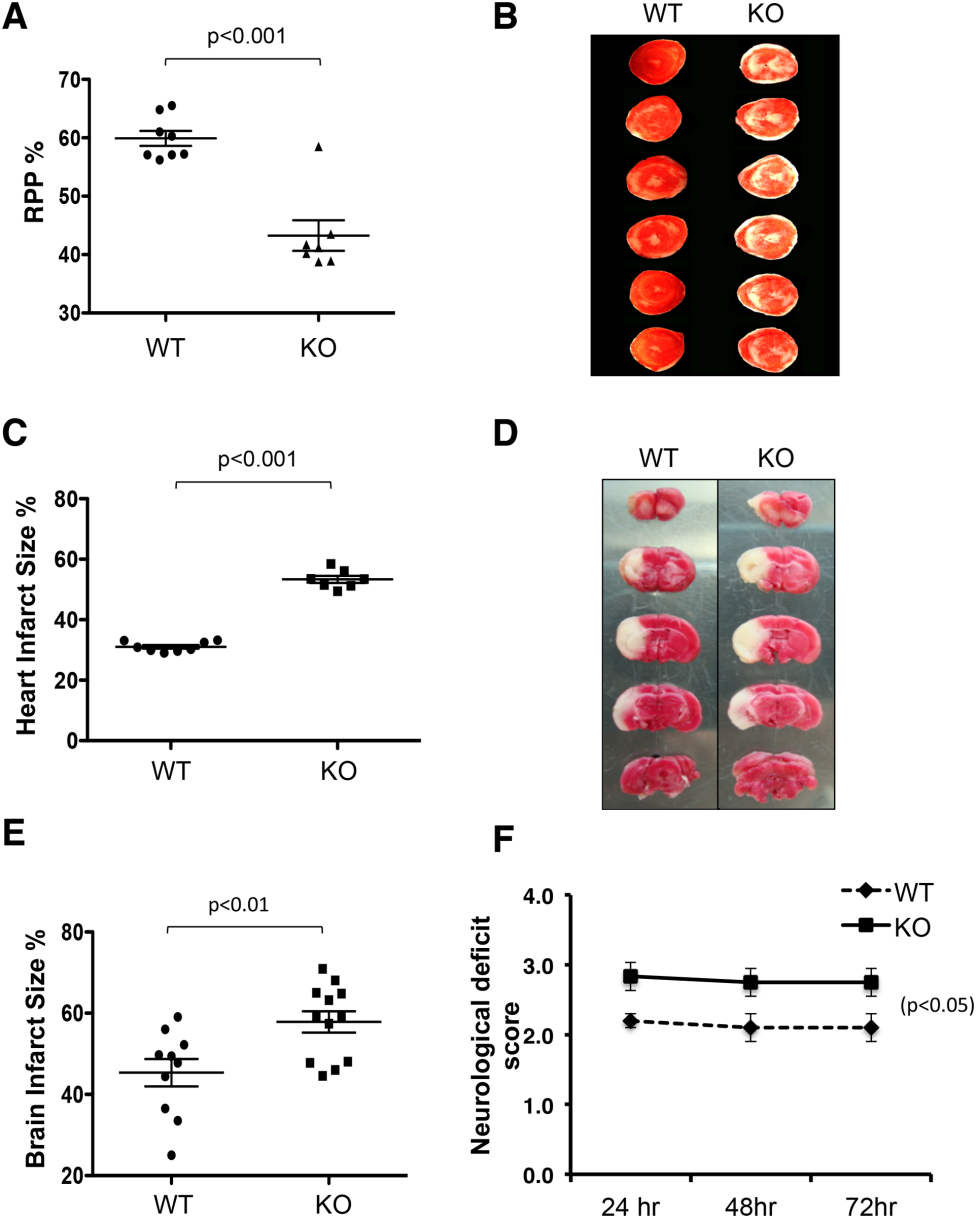
PGAM5 protects hearts (A-C) and brain (D-F) from ischemia-reperfusion (I/R) injury induced cell death. I/R-induced myocardial damage was evaluated based on RPP values (A) and heart infarct size (B,C). Pgam5 WT mice n = 9, KO mice n = 10. (*p* <0.01 by Student *t*-test; Pgam5 KO mice exhibit increased infarct volumes (D, E). *p*<0.01 by Student *t*-test. Pgam5 WT mice n = 13, KO mice n = 16 after I/R induced brain damage and (F) greater neurological deficit scores. *p*<0.05 by Student *t*-test.

Experimental ischemia/reperfusion (I/R) in the heart and brain are models for myocardial infarction and stroke, respectively, which are the two most common causes of non-infectious morbidity and mortality worldwide [24]. When the blood supply carrying oxygen and nutrients is interrupted by thrombosis and then restored in the tissue such as the heart or brain, the damaged mitochondria produce excessive ROS, causing oxidative injury and necroptosis [21, 24, 25]. We explanted hearts from both Pgam5 WT and KO mice; induced ischemia for 25 minutes followed by 90 minutes of reperfusion, and evaluated hemodynamic function as well as histologically apparent infarct size [18, 26]. Before I/R, the basal hemodynamic parameters for hearts of Pgam5 KO mice were the same as those from WT mice, indicating no constitutive effect of PGAM5 deficiency. However, following I/R we found a significantly reduced rate pressure product (RPP) in Pgam5 KO compared to WT hearts indicating inferior heart function (Fig 1A). Staining for live cells with intact mitochondria using triphenyl-tetrazolium chloride (TTC), a dye oxidized by mitochondrial dehydrogenase to yield the red product formazan, revealed substantially larger unstained infarct areas (white) in Pgam5 KO compared with WT I/R hearts (Fig 1B and 1C). This suggested increased cell necrosis in the absence of PGAM5. To examine the role of PGAM5 in the brain following I/R, we subjected WT and Pgam5 KO mice to middle cerebral artery occlusion (MCAO) for 1 hour [26–28]. After 72 hours of reperfusion, we found that the infarct size in the Pgam5 KO brain was also significantly increased compared to WT brain (Fig 1D and 1E). Pgam5 KO mice showed greater neurological deficits in limb movement as a consequence of MCAO injury (Fig 1F). Thus a protective effect of PGAM5 was evident despite the fact that a much longer time course of I/R was used in the MCAO studies. In conclusion, these observations suggest a new model for PGAM5 function as a protective mediator of necroptosis in vivo, which is in contrary to its roles in tumor cell lines [12]. We found that both myocardial and cerebral necrosis after I/R are more severe in Pgam5 KO mice, indicating that *in vivo* PGAM5 is important in protecting from I/R induced cell necroptosis.

### PGAM5 protects cells from necroptosis

**Fig 2:**
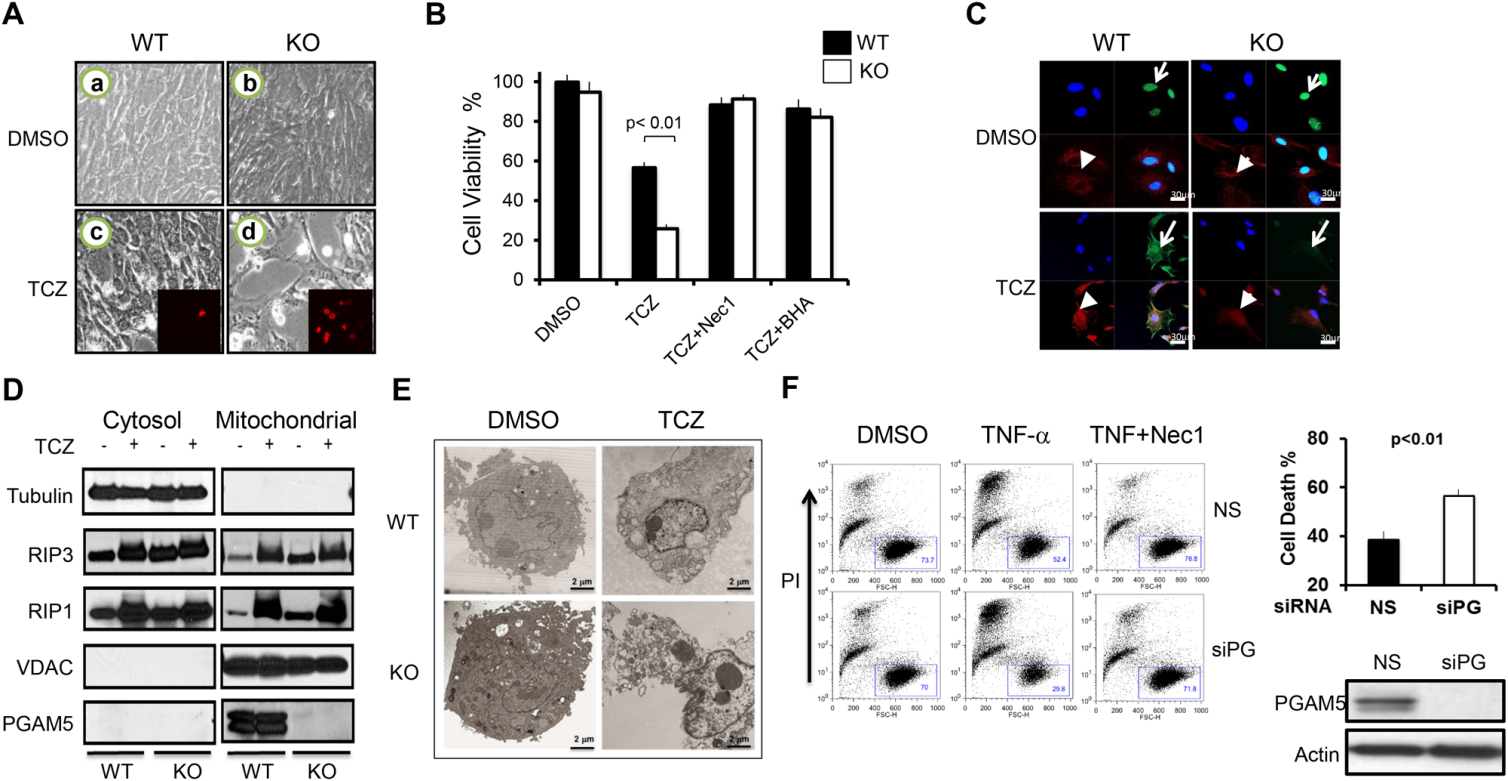
PGAM5 deficiency exacerbates necroptosis. (A) Phase contrast photomicrographs of WT or KO MEFs treated with DMSO (a, b) or TCZ (c, d) for 24 hr. Cell viability was evaluated by the MTT assay, and propidium iodide staining (inset c, d). (B) Cell viability results for WT (black) and KO (white) MEFs, which were treated with TCZ with or without 50 μM Nec1 or 100 μM BHA; (C) WT and Pgam5 KO MEFs were treated with DMSO or TCZ for 6 hr and then stained with the mitochondria membrane potential dependent dye DAPI (blue), anti-HMGB1 (green) and Mitotracker (red); (D) WT and Pgam5 KO MEFs were treated with TCZ for 3 hrs, and followed by purification of mitochondria. Western blots with the indicated antibodies are shown. Tubulin and VDAC are loading controls. (E) Representative TEM micrographs of necrotic WT and Pgam5 KO MEFs treated with TCZ or DMSO vehicle for 12 hrs. (F) Jurkat I2.1 (Caspase 8 deficient) cells transfected with nonspecific (NS) or PGAM5 (siPG) siRNAs were treated with 0.5 ug/ml human TNF-α for 24 hrs with or without necrostatin (Nec1), then cell death was quantified by taking the percentage outside of the life gate (indicated by the box using dot plots with PI staining on the y-axis and forward scatter on the x-axis (left panels). Quantification is shown in a bar graph (right upper panel) and the quality of the knockdown shown by Western blot (right lower panel).

We generated Pgam5 Wild type (WT) and knockout (KO) mouse embryonic fibroblasts MEFs at embryonic day 12.5 (E12.5) and compared their fate after treatment with a combination of TNF-α, cycloheximide (CHX), and z-VAD-fmk (TCZ) (Fig 2). TCZ triggers ROS-dependent necroptosis by signaling through the TNF receptor signaling, when CHX blocks translation of pro-survival molecules and z-VAD-fmk prevents apoptosis by inhibiting caspases [8–11, 29, 30]. We found that non-transformed Pgam5 KO MEFs underwent substantially more cell death than WT MEFs (Fig 2A and 2B).

Several lines of evidence verified that the enhanced cell death occurring in PGAM5-deficient MEFs was necroptosis. These include the early incorporation of propidium iodide indicating membrane breakdown (Fig 2A, panels c and d) and the blockade of TCZ-induced cell death of MEFs by the RIP1 kinase inhibitor necrostatin-1 (Nec1) [31] or the reactive oxygen species (ROS) scavenger butylated hydroxyanisole (BHA) [29] (Fig 2B). After 6 hours of necroptotic stimulation, we also observed the cytosolic staining of nuclear protein High Mobility Group Box 1 (HMGB1), which is a well-established necroptosis marker and selectively translocates to the cytoplasm during the early stages of necroptosis [32, 33] (Fig 2C, Green, indicated by white arrow). Meanwhile, using a specific, mitochondrial membrane potential dependent dye, Mitotracker Red, we found that necrotic stimulation disrupted the mitochondria membrane potential, as evidenced by the loss of mitochondria network structure after TCZ treatment (Fig 2C, Red, indicated by arrow head). Previously it was proposed that PGAM5 recruits RIP1/RIP3 to the mitochondria during necroptosis [34]. Since this seemed inconsistent with our *in vivo* results, we examined this question by purifying mitochondria and assessing the associated proteins before and after necroptosis induction. We found that in both Pgam5 WT and KO MEFs, TCZ stimulation increased RIP1 and RIP3 accumulation on mitochondria (Fig 2D). Thus, during necroptosis the RIP1/RIP3 complex localizes to the mitochondria independently of the presence of PGAM5. Transmission electron microscopy (TEM) analysis demonstrated the classical morphological features of cell necrosis including loss of plasma membrane integrity and dissolution of cellular structures confirming that necrosis had occurred under these conditions in both WT and KO cells (Fig 2E).

In contrast to human WT Jurkat T cells, the FADD-deficient Jurkat T cell line I2.1 undergoes specific necroptosis in response to TNF-α stimulation without CHX [34]. We found that siRNA knockdown of PGAM5 in I2.1 cells enhanced TNF-α-induced cell necrosis (Fig 2F). From these data, we conclude that PGAM5 reduces necroptosis in different cell types which complicates previous models suggesting a central role in mediating RIP1/RIP3-dependent necroptotic pathway. Rather, PGAM5 appears to exert a cytoprotective function rather than directly execute necroptosis.

### PGAM5 promotes mitophagy during necroptosis

**Fig 3:**
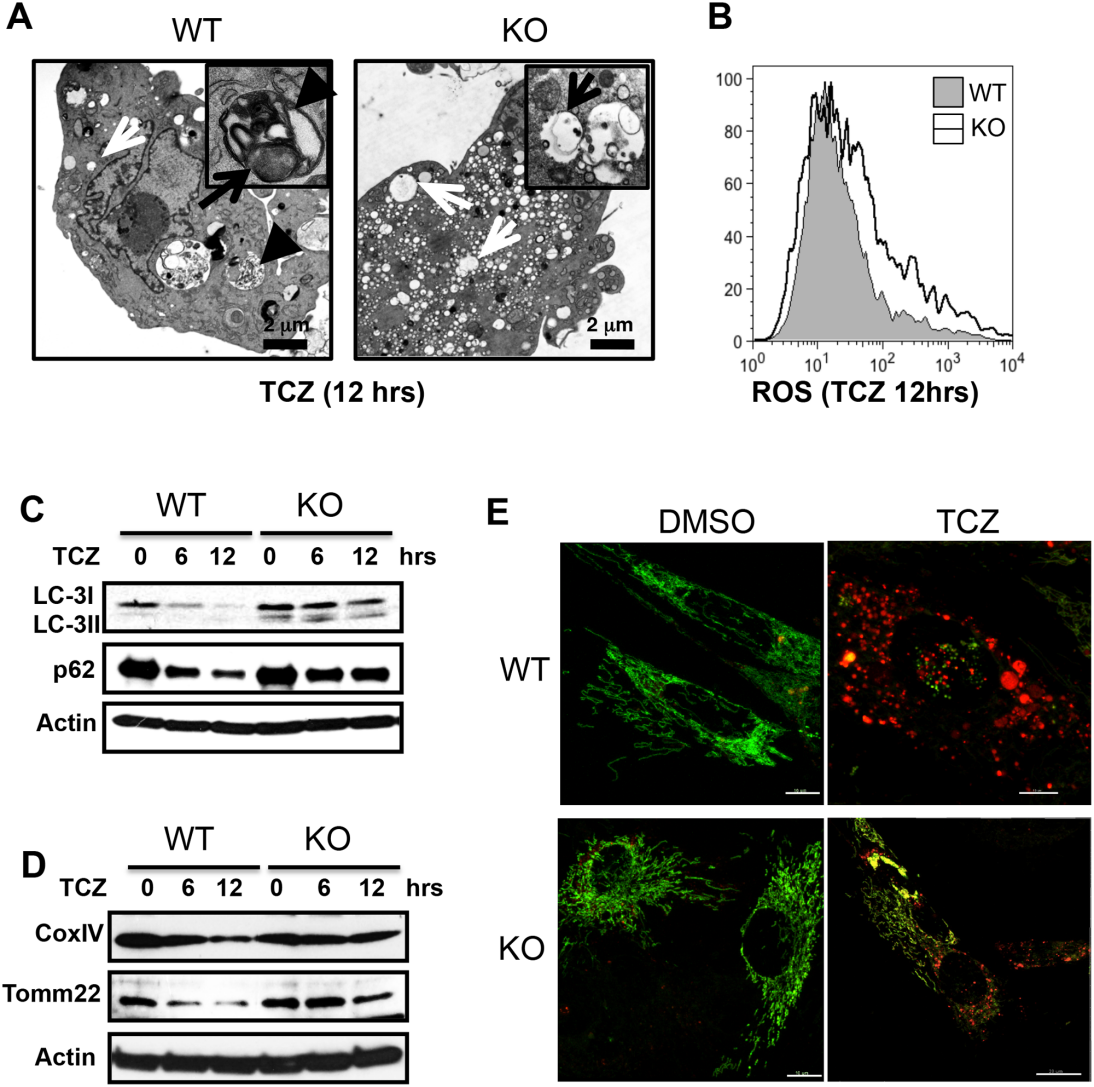
PGAM5 is required for the mitophagy during cell necroptosis. (A) TEM micrographs of WT and Pgam5 KO MEFs before and after TCZ treatment. Black arrowheads indicate double-membrane structures with encapsulated mitochondria. The black arrow indicates the mitochondria. The white arrows indicate empty vacuoles. (B) Intracellular ROS level was measured by ROS dye CM-H2CFDA for 30 minutes and analyzed by flow cytometry. WT and Pgam5 KO MEFs were treated with TCZ for the indicated time and proteins from cytosolic part (C) or total cell lysates (D) were blotted for the indicated proteins. (E) Confocal photomicrographs of mtKeima-transduced WT and Pgam5 KO MEFs treated with TCZ for 12 hrs after excitation at 450 nm and at 560 nm.

To elucidate the cytoprotective mechanism of PGAM5, we analyzed mitochondria in WT and KO MEFs after necroptotic stimulation by TEM. When damaged mitochondria cannot be cleared by mitophagy, excessive ROS can result in necroptosis [3, 21, 25]. After TCZ stimulation for 24 hours, we found that both Pgam5 WT and KO preparations contained numerous vesicles displaying typical necroptotic phenotypes. WT cells exhibited double-membrane autophagosomes with encapsulated mitochondria (Fig 3A, left panel). By contrast, Pgam5 KO cells had a large number of irregular single membrane cytosolic vacuoles that appeared to exclude mitochondria suggesting that mitophagy was defective (Fig 3A, right panel). TCZ induction of intracellular ROS was greater in KO cells relative to WT cells (Fig 3B). We next performed biochemical analyses of mitophagy and found that although the microtubule-associated protein 1A/1B light chain 3 (LC-3) was processed from LC-3I to LC-3II in both WT and Pgam5 KO MEF cells after TCZ treatment, the autophagic removal of LC-3 and cytosolic sequestosome 1/p62, was less efficient in Pgam5 KO MEFs (Fig 3C) [35]. With longer stimulation (12 h), Pgam5 KO MEFs showed impaired mitochondrial clearance as compared to the WT MEFs, evidenced by the persistence of Tomm22 (mitochondrial outer membrane protein) and COX IV (mitochondrial inner membrane protein) in total cell lysates (Fig 3D). We directly visualized mitophagy by using mt-Keima, a mitochondrion-targeted fluorescent protein marker resistant to lysosomal proteases whose excitation fluorescence (λ_ex_) occurs at 450 nm (indicated by green fluorescence) at cytosolic neutral pH and 550 nm (indicated by red fluorescence) at acid pH, thereby providing a readout for mitochondrial delivery to acidic lysosomes [36]. We observed that TCZ treatment of WT MEFs induced a dramatic shift of mt-Keima from an evenly distributed green mitochondrial appearance to a bright red punctate appearance, indicating relocation of mitochondria to acidic autolysosomes by mitophagy (Fig 3E, top panels). By contrast, Pgam5 KO cells exhibited very little red punctate staining, indicating defective mitophagic delivery of damaged mitochondria to lysosomes (Fig 3E, bottom panels). Hence, PGAM5 appears to be indispensable for the process of mitophagy, which aids in preventing necroptosis by clearing ROS-producing unhealthy mitochondria [21, 37].

### PGAM5 prevents necroptosis through PINK1-mediated mitophagy

**Fig 4:**
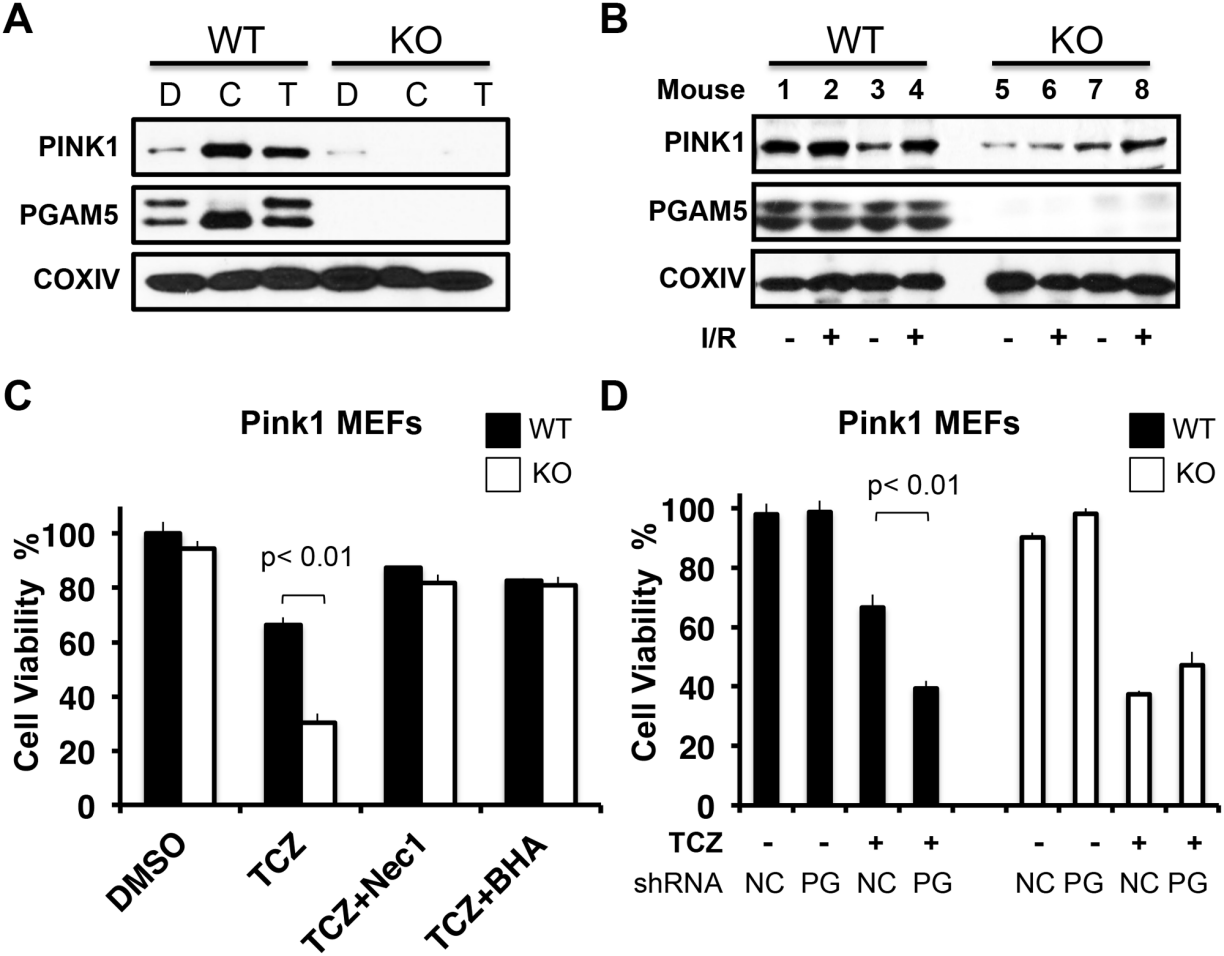
PGAM5 stabilizes PINK1 to protect cell from necroptosis. (A) Mitochondrial extracts of WT and Pgam5 KO MEFs treated with DMSO (D), CCCP(C), or TCZ (T) for 3 hours were analyzed by immunoblot as above. (B) PINK1 immunoblot of mitochondrial fractions from WT and KO hearts subjected to I/R (+) or perfusion only (-). (C) Cell viability of Pink1 WT/KO MEFs was quantified by MTT assay. Black bars represent WT controls and white bars the respective KO. Cells were treated with TCZ, TCZ plus Nec1, or TCZ plus BHA for 12 hours. (D) Cell viability of WT and Pink1 KO MEF cells was transduced with scrambled (NC) or Pgam5-specific shRNA (PG) lentiviruses, treated with TCZ, and then tested for viability by the MTT assay at indicated treatments. *p* <0.01 by Student *t*-test.

Mitophagy is essential to maintain intracellular mitochondria homeostasis and decrease levels of ROS that can cause necrotic cell death [38]. Several key regulators have been identified as important for mitophagy including PINK1 and Parkin [39–41]. We therefore investigated the effect of necroptotic stimuli on PINK1 stabilization on the mitochondria. Carbonyl cyanide m-chlorophenylhydrazone (CCCP), which can disrupt mitochondrial membrane potential and stabilize PINK1, was also included as control treatment [41]. We found that either TCZ- or CCCP-induced PINK1 stabilization in WT MEFs was completely defective in PGAM5-deficient cells (Fig 4A). We also extracted mitochondria from isolated mouse hearts subjected to I/R-induced necroptosis. Consistent with our *in vitro* studies, we found that I/R caused more mitochondrial PINK1 stabilization on cardiac mitochondria in WT mice, while KO mice had a marked deficit of stabilized mitochondrial PINK1 (Fig 4B). Therefore, PGAM5 promotes PINK1-dependent mitophagy during cell necroptosis *in vitro* and *in vivo*.

To further investigate the role of mitophagy in limiting necroptosis, we treated PINK1 deficient MEFs with TCZ to induce cell death. As we observed for Pgam5 KO cells, necroptotic cell death was augmented in Pink1 KO compared to WT MEFs (Fig 4C and S2 Fig). Both Nec1 and BHA blocked TCZ-induced cell death in both Pink1 KO and WT MEFs, consistent with the idea that reduced cell viability was due to compromised mitophagy during cell necroptosis (Fig 4C). Furthermore, lentiviral expression of PINK1 restored the level of necroptosis induced by TCZ to the same level as WT cells (Data in S1 Fig). PGAM5 is likely epistatic to PINK1 function in the mitophagy pathway because no further increase in necroptosis was observed when Pgam5 was knocked down (KD) by shRNA in Pink1 KO MEFs despite a clear effect of the KD in Pink1 WT cells (Fig 4D). Taken together, these data confirm that PGAM5, cooperating with PINK1, protects cells from necroptosis by promoting mitophagy.

### PGAM5/PINK1 mitophagic pathway is default in HT-29 cells

**Fig 5:**
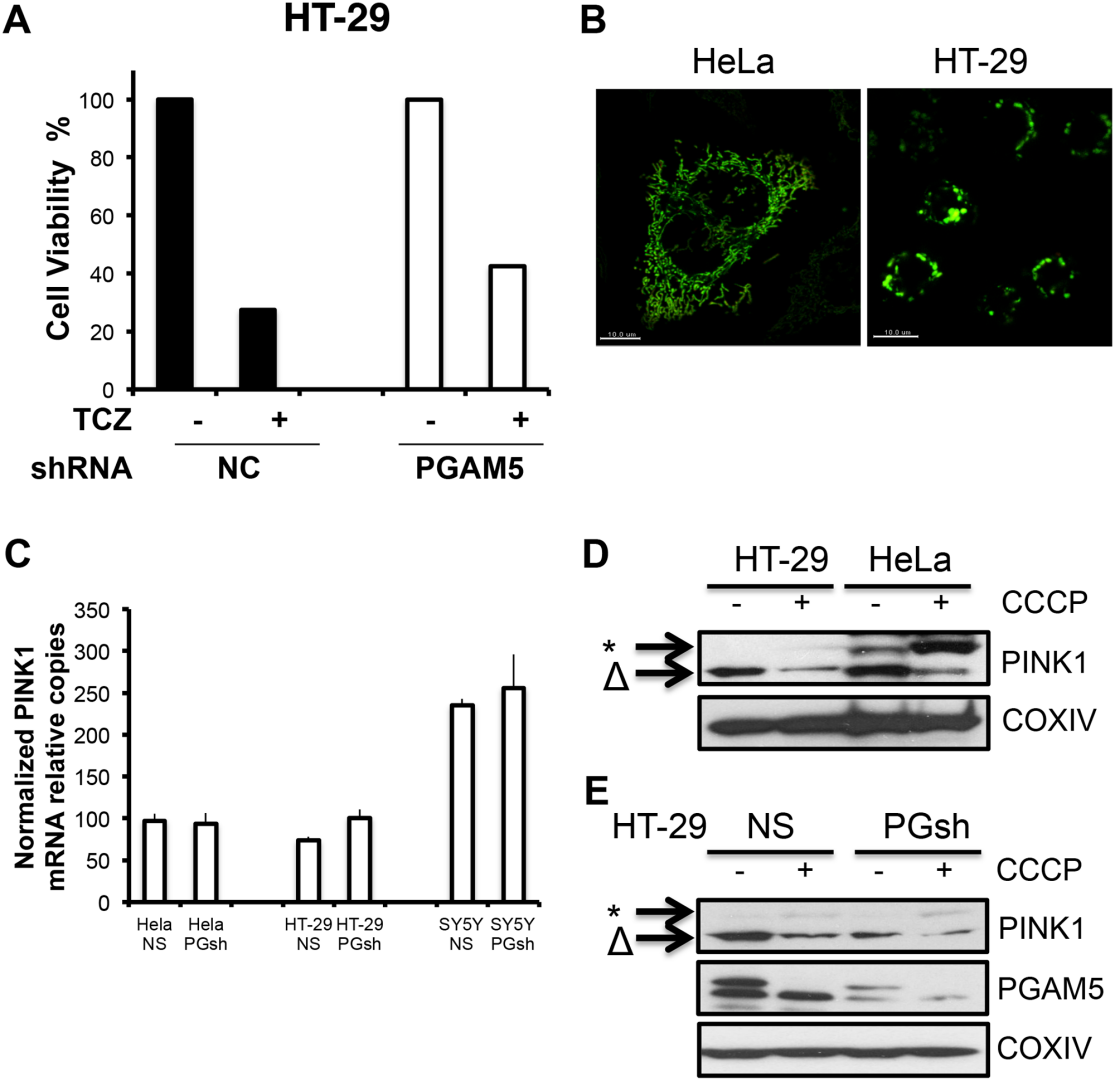
PGAM5 fails to stabilize PINK1 in HT-29 cells. (A) HT-29 cells transduced with shRNA against PGAM5 were treated with TCZ to induce necroptosis. Cell viability was evaluated by the MTT assay. (B) Mitochondria in HeLa cells and HT-29 cells were stained with anti-Tomm20 and mitochondrial morphology was evaluated by confocal. (C) PGAM5 was knocked down by lentiviral shRNA in HeLa, HT-29 and SY5Y cell lines. Then PINK1 mRNA in the control (NS) and Knock-down cells (PGsh) were quantified by RT-PCR. (D) HT-29 and HeLa cells were treated with CCCP for 3 hours and PINK1 was detected by western blot. * indicated full length PINK1 and Δ indicated cleaved PINK1.(E)HT-29 control (NS) and PGAM5 knock-down cells (PGsh) were treated with CCCP for 3 hours followed by western blot for detecting indicated proteins.

The model for PGAM5 as a convergence point in promoting necroptosis arose from experiments using the human colorectal cancer cell line, HT-29 cells [12]. We therefore knocked down PGAM5 with lentiviral shRNA, and only observed slight, if any, protection from TCZ-induced necroptosis (Fig 5A). To address this discrepancy, we evaluated mitochondria in HT-29 cells. Compared to other cell types used in this study, HeLa and MEFs, the mitochondria in HT-29 cells appear to be a strikingly abnormal aggregated morphology suggesting defective organelle function (Fig 5B). Moreover, after CCCP treatment, HT-29 cells failed to stabilize the full length PINK1 on the mitochondria despite comparable PINK1 mRNA in both HT-29 and HeLa cells (Fig 5C and 5D). By comparison, HeLa cells, which were used as a positive control, exhibited effective full-length PINK1 stabilization (Fig 5D). Thus, we conclude that silencing the endogenous PGAM5 in HT-29 cells did not influence PINK1-mediated mitophagy because there is an underlying mitochondrial abnormality (Fig 5E).

## Discussion

Mitochondria have a physiological role in regulating cell death both by apoptosis as well as necroptosis pathways. In response to cellular stress, mitochondria produce ROS and other pro-death mediators [21, 38, 42]. The molecular basis of necroptosis was recently established by elucidating the roles of RIP1 and RIP3 in increasing the mitochondrial production of reactive oxygen species (ROS) levels [21, 25]. Previous studies have proposed that PGAM5, a mitochondrial membrane protein recruits RIP1/RIP3 to the mitochondria to cause cell necroptosis [12]. We find that although the RIP1/RIP3 complex was enriched on the mitochondria during the necroptotic stimulation, PGAM5 has very little influence on RIP1/3 recruitment because this process occurs in cells genetically deficient in PGAM5. Moreover, we show that PGAM5 has a prominent necroptosis protective function, both *in vitro* and *in vivo*. This appears to be due to the ability of PGAM5 to promote PINK1 dependent mitophagy. Our new data are also consistent with our previous observations that Pgam5 knockout mice manifest a Parkinson’s disease phenotype due to dopamine neuron degeneration [18]. We have also deduced that the discrepancy between our results and the previous report [12] is due to cell type variation. HT-29 cell is a colon adenocarcinoma tumor cell line with hypertriploid chromosome, abnormal mitochondria, and a p53 gene mutation [43], and therefore may present an abnormal picture of physiological cell death pathways.

Our data demonstrate that during necroptosis, ROS overproduction can induce mitophagy, a selective form of autophagy that targets unhealthy mitochondria for lysosomal recycling. We have shown that the mitophagy process is PGAM5-dependent and also cytoprotective against necroptosis. Very few regulators of mitophagy have been identified despite the importance of defective mitophagy in association with a variety of diseases, including Parkinson’s disease and ischemia injury in myocardial infarction and stroke [38, 44]. ROS generation during I/R injury causes oxidative damage and cell necrosis, which have been attributed to damaged mitochondria [24, 38, 44]. Therefore, understanding the detailed mechanism of mitophagy remains an important goal for improving the diagnosis and treatment of diseases involving mitochondria and necroptosis. Our studies unveil a new function of the prominent mitochondrial regulatory protein PGAM5. In response to ROS, such as those generated during I/R injury, PGAM5 can play important cardioprotective and neuroprotective roles by limiting necrosis most likely through enhancing mitophagy. Our data support the previous reports showing that PINK1 and Parkin, both essential for mitophagy, can also protect hearts from I/R injury by enhancing the removal of damaged mitochondria [45, 46]. Hence, further investigation of PGAM5 may lead to new therapeutic targets and a better understanding of diseases characterized by mitochondrial dysfunction and ROS-mediated cellular necroptosis, including ischemic cardiovascular/myocardial infarction and cerebrovascular/stroke and, potentially, neurodegenerative disorders such as Parkinson’s disease.

## Materials and Methods

### Mice and Cells

C57BL/6 mice and Pgam5 KO mice in C57BL/6 background were maintained and treated in accordance with NIAID Animal Care and Use Committee guidelines [18]. Mice were sacrificed by CO2 inhalation for sample collection. MEF cells were prepared from day 12.5 embryos and cultured in DMEM/F12 full media (FM) (FM: 10% fetal bovine serum, 4 mM L-glutamine, 100 IU/mL penicillin, and 100 mg/mL streptomycin) plus non-essential amino acids. PINK1-knockout MEFs were kindly provided by Dr. Zhuohua Zhang (Sanford Burnham Medical Research Institute).

### Antibodies

PGAM5 antibody was made against the peptide, CGSLEKDRTLTPLGR by Genscript (NJ, USA). Commercial antibodies were RIP1 (BD Bioscience); RIP3 (eBioscience); PINK1 and PARL (Sigma); and COXIV, VDAC, Actin, HSP60, HMGB1 and tubulin antibodies (Cell Signaling Technology).

### Lentiviral, shRNA-mediated knockdown and cDNA overexpression

pLKO.1 Pgam5 shRNA plasmids were purchased from Thermo Scientific. MEF cells were infected with lentivirus encoding shRNA against PGAM5 for 24 hours before selection with 10 μg/mL puromycin according to manufacturer’s specifications. The PGAM5-GFP sequence was cloned into bicistronic lentiviral vectors (Abmgood). GFP control virus and PGAM5-GFP lentivirus were used to transduce MEF cells followed by puromycin selection as above.

### Cell viability assay

MEFs were treated with 25 ng/mL mTNF-α (Biolegend), 50 μM z-VAD (Enzo Lifescience), and 1 μg/mL CHX (Sigma) for 24 hours and cell viability was quantified by CellTiter 96 aqueous non-radioactive assays MTT (Promega) according to the manufacturer’s protocol. I2.1 Jurkat cells were treated with 0.5 ng/mL hTNF-α (R&D) for 24 hours then stained with propidium iodide and analyzed by flow cytometry.

### Western blot

Cells were collected and lysed in buffer (20 mM Tris/HCl, pH 7.2, 0.5% (v/l) Nonidet P-40, 300 mM NaCl, 3 mM EDTA, 3 mM EGTA, 2 mM dithiothreitol, 2 mM PMSF, 100 μM leupeptin, 10 μM bestatin, 10 μM pepstatin and 2 μg/mL of aprotinin). Supernatants were collected after centrifugation at 10,000 x g for 20 min at 4°C, separated by 4–20% SDS-PAGE and analyzed by immunoblot. Cells and tissue mitochondria were purified by using Qproteome mitochondria isolation kit (Qiagen). The mitochondria pellet was then lysed in 2X SDS loading buffer and loaded onto SDS-PAGE gels.

### Live cell imaging

MEFs stably transduced with lentivirus encoding mitochondria-targeted Keima protein (mtKeima) were cultured in glass-bottom dishes (MatTek). Cells were treated with CCCP for 24 hr or with TCZ for 12 hr and then imaged using the Leica SP5 confocal system. Data were analyzed using Imaris software.

### Langendorff heart ischemia reperfusion model for myocardial infarction

These experiments were performed with gender and age-matched littermates. All animal experiments were performed in accordance and with approval from the NHLBI Animal Care and Use Committee. Hearts from WT or Pgam5 KO littermates were isolated and perfused using a Langendorff apparatus with Krebs-Heinseleit (KH) buffer for 20 min before being subjected to 25 min of no-flow ischemia. Functional recovery of left ventricular developed pressure (LVDP) and rate pressure product (RPP, product of LVDP and heart rate) was measured following 90 min of reperfusion and expressed as a percentage of the initial RPP prior to ischemia as previously described. At the end of 90 min of reperfusion, hearts were perfused with KH buffer containing 1% (w/v) 2,3,5-triphenyltetrazolium chloride (TTC) and then incubated in TTC at 37°C for 15 min, followed by fixation in 10% (w/v) formaldehyde. Hearts were sliced into cross-sections and infarct size was expressed as the percentage of total area of cross-sectional slices through the ventricles.

### Focal middle carotid artery occlusion/reperfusion (MCAO/R) ischemic stroke model

The MCAO/R model was performed using the intraluminal suture technique described previously. Briefly, Mice were anesthetized with isoflurane, a midline neck incision was made and the right external carotid and pterygopalatine arteries were ligated with 6–0 silk thread. The internal carotid artery was occluded at the peripheral site of the bifurcation of the internal carotid artery (ICA) and the pterygopalatine artery with a small clip and the common carotid artery (CCA) were ligated with 6–0 silk thread. The external carotid artery (ECA) was cut, and 6–0 nylon monofilament coated with a mixture of silicone resin was advanced into the middle cerebral artery (MCA) until resistance was felt. The nylon thread and the CCA ligature were removed after 1 h of occlusion to initiate reperfusion. In a separate set of experiments anesthetized animals from all groups (4 mice per group) underwent cerebral blood flow (CBF) measurements using a laser Doppler perfusion monitor. All CBF measurements were conducted with the mouse supported in a plastic frame with the probe placed in the region of cerebral cortex perfused by the MCA. There were no significant differences in global CBF between WT and Pgam5 KO mice, before, during or after MCA occlusion. The functional consequences of MCAO/R were evaluated in a masked fashion using a five-point score (0, no deficit; 1, failure to extend left paw; 2, circling to the left; 3, falling to the left; 4, unable to the walk spontaneously). At 72 h of reperfusion, the mice were sacrificed and the brains were placed into PBS (4°C) for 15 min, and 2-mm coronal sections prepared. The brain sections were stained for infarct area with 2% TTC in phosphate buffer at 37°C for 20 min and then immediately fixed in 10% formalin overnight. Digitized images were used to outline the borders of the infarct in each brain slice and the area was quantified by NIH image 6.1 software. Infarct area was determined by subtracting the area of the non-infarcted ischemic hemisphere from intact non-ischemic hemisphere. Percent infarct volume was calculated by dividing the sum of the infarct area by the total of that of non-ischemic hemisphere to correct for tissue edema.

## Supporting Information

S1 Fig Re-introduced PINK1 in Pink1 KO MEFs protect cells from necroptosis. Immortalized Pink1 KO MEFs were transduced with lentivirus expressing Wide type PINK1 protein, or control vector. Transduced KO MEFs and Pink1 WT MEFs were then treated with TNF-α, z-VAD as well as CHX as mentioned in the manuscript (Necroptosis inhibitor Necrostain 1 (Nec1) was also involved as an inhibitor for necroptosis). Finally, cell viability was evaluated by MTT assay.
(DOCX)

S2 Fig LDH cytotoxicity assay for the TCZ induced necroptosis in Pink1 WT and KO MEFs. Pink1 WT and KO MEFs (50,000 cells/well) were plated in 24-well plate. 12 hours later, cells were treated with TNF-α, z-VAD and Cycloheximide as mentioned in the manuscript. LDH cytotoxicity was measured by using the Pierce LDH cytotoxity Assay Kit.
(DOCX)
