# Supplementary material for: Mitochondrial Protein PGAM5 Regulates Mitophagic Protection against Cell Necroptosis
Source: PLoS One. 2016 Jan 25;11(1):e0147792. doi: 10.1371/journal.pone.0147792 (PMC4725845; doi:10.1371/journal.pone.0147792)
Supplement: S1 Fig — Immortalized Pink1 KO MEFs were transduced with lentivirus expressing Wide type PINK1 protein, or control vector. Transduced KO MEFs and Pink1 WT MEFs were then treated with TNF-α, z-VAD as well as CHX as mentioned in the manuscript (Necroptosis inhibitor Necrostain 1 (Nec1) was also involved as an inhibitor for necroptosis). Finally, cell viability was evaluated by MTT assay. (DOCX) [file pone.0147792.s001.docx]

**S1 Figure. Re-introduced PINK1 in Pink1 KO MEFs protect cells from necroptosis.**



Immortalized Pink1 KO MEFs were transduced with lentivirus expressing Wide type PINK1 protein, or control vector. Transduced KO MEFs and Pink1 WT MEFs were then treated with TNF-a, z-VAD as well as CHX as mentioned in the manuscript (Necroptosis inhibitor Necrostain 1 (Nec1) was also involved as an inhibitor for necroptosis). Finally, cell viability was evaluated by MTT assay.
