# Supplementary material for: Mitochondrial Protein PGAM5 Regulates Mitophagic Protection against Cell Necroptosis
Source: PLoS One. 2016 Jan 25;11(1):e0147792. doi: 10.1371/journal.pone.0147792 (PMC4725845; doi:10.1371/journal.pone.0147792)
Supplement: S2 Fig — Pink1 WT and KO MEFs (50,000 cells/well) were plated in 24-well plate. 12 hours later, cells were treated with TNF-α, z-VAD and Cycloheximide as mentioned in the manuscript. LDH cytotoxicity was measured by using the Pierce LDH cytotoxity Assay Kit. (DOCX) [file pone.0147792.s002.docx]

**S2 Figure. LDH cytotoxicity assay for the TCZ induced necroptosis in Pink1 WT and KO MEFs.**

*

*

Pink1 WT and KO MEFs (50,000 cells/well) were plated in 24-well plate. 12 hours later, cells were treated with TNF-a, z-VAD and Cycloheximide as mentioned in the manuscript. LDH cytotoxicity was measured by using the Pierce LDH cytotoxity Assay Kit.
